# Supplementary material for: Huntingtin phosphorylation governs BDNF homeostasis and improves the phenotype of Mecp2 knockout mice
Source: EMBO Mol Med. 2020 Jan 8;12(2):e10889. doi: 10.15252/emmm.201910889 (PMC7005633; doi:10.15252/emmm.201910889)
Supplement: Supplementary file 1 — Appendix [file EMMM-12-e10889-s001.pdf]

Huntingtin phosphorylation governs BDNF homeostasis and improves the phenotype of Mecp2 knockout mice

Ehinger et al, 2019

Table of contents :

- **Appendix Table S1:** The modified SHIRPA primary screen in WT, HTT_{SD} and HTT_{SA} mice.

- **Appendix Table S2:** Statistical information for main figures

- **Appendix Table S3:** Statistical information for supplementary figures

Appendix Table S1

	Tests	WT (n=11)	HTT_{SA} (n=12)	HTT_{SD} (n= 11)
section 1	**In the viewing Jar**			
	Coat color	Normal: 11 (100%)	Normal: 12 (100%)	Normal: 11 (100%)
	Hair length	Normal: 11 (100%)	Normal: 12 (100%)	Normal: 11 (100%)
	Respiration rate	Normal: 11 (100%)	Normal: 12 (100%)	Normal: 11 (100%)
	Tremor	None: 11 (100%)	None: 12 (100%)	None: 11 (100%)
	Body position	Sitting or standing: 11 (100%)	Sitting or standing: 12 (100%)	Sitting or standing: 11 (100%)
	Spontaneous activity	Vigorous scratch, groom, moderate movement: 11 (100%)	Vigorous scratch, groom, moderate movement: 12 (100%)	Vigorous scratch, groom, moderate movement: 11(100%)
	Defecation	None: 10 (91%)	None: 11 (92%)	None: 7 (64%)
		Done: 1 (9%)	Done: 1 (8%)	Done: 4 (36%)
	Urination	None: 11 (100%)	None: 9 (75%)	None: 9 (82%)
		Done: 0 (0%)	Done: 3 (25%)	Done: 2 (18%)
section 2	**In the Arena**			
	Elapsed time before the mouse starts to move (s)	Less than 3s: 11 (100%)	Less than 3s: 12 (100%)	Less than 3s: 11 (100%)
	Transfer arousal	Brief freeze (few sec), then	Brief freeze (few sec), then	Brief freeze (few sec), then
		active movement: 11 (100%)	active movement: 12 (100%)	active movement: 11 (100%)
	Locomotor activity	Average number of squares entered over 1 min exploration: 37 ± 1.859	Average number of squares entered over 1 min exploration: 38.33 ± 1.707	Average number of squares entered over 1 min exploration: 27.55 ± 2.644
	Palpebral Closure	Eyes wide open: 11 (100%)	Eyes wide open: 12 (100%)	Eyes wide open: 11 (100%)
	Piloerection	None: 11 (100%)	None: 12 (100%)	None: 11 (100%)
	Gait	Normal: 11 (100%)	Normal: 11 (92%)	Normal: 11 (100%)
		Fluid but abnormal: 0 (0%)	Fluid but abnormal: 1 (8%)	Fluid but abnormal: 0 (0%)
	Startle Response	Preyer reflex (backwards	Preyer reflex (backwards	Preyer reflex (backwards
		flick of pinnae): 11 (100%)	flick of pinnae): 12 (100%)	flick of pinnae): 11 (100%)
	Pelvic Elevation	Normal (3mm elevation): 11 (100%)	Normal (3mm elevation): 12 (100%)	Normal (3mm elevation): 11 (100%)
	Tail Elevation	Horizontally extended: 11 (100%)	Horizontally extended: 12 (100%)	Horizontally extended: 11 (100%)
	Touch Escape	Mild (escape response	Mild (escape response	Mild (escape response
		to firm stroke):11 (100%)	to firm stroke): 12 (100%)	to firm stroke): 11 (100%)
	Tail morphology	Normal: 11 (100%)	Normal: 12 (100%)	Normal: 10 (91%)
		Kinky: 0 (0%)	Kinky: 0 (0%)	Kinky: 1 (9%)
	Convulsions	Phenotype: 0 (0%)	Phenotype: 0 (0%)	Phenotype: 0 (0%)
section 3	**On or Above the arena**			
	Wire manoeuver (Horizontal bar)	Active grip with hindlegs (5sec): 5 (45%)	Active grip with hindlegs (5sec): 3 (25%)	Active grip with hindlegs (5sec): 2 (18%)
		Difficulty to grasp with hindlegs: 2 (18%)	Difficulty to grasp with hindlegs: 3 (25%)	Difficulty to grasp with hindlegs: 4 (36%)
		Unable to grasp with hindlegs: 1 (9%)	Unable to grasp with hindlegs: 1 (8%)	Unable to grasp with hindlegs: 0 (0%)
		Unable to lift hindlegs, falls within seconds: 3 (27%)	Unable to lift hindlegs, falls within seconds: 5 (42%)	Unable to lift hindlegs, falls within seconds: 5 (45%)
		Falls immediately: 0 (0%)	Falls immediately: 0 (0%)	Falls immediately: 0 (0%)
	Negative geotaxis (vertical grids)	Turns and climbs the grid: 11 (100%)	Turns and climbs the grid: 12 (100%)	Turns and climbs the grid: 11 (100%)
	Positional Passivity : struggles when held by tail	Yes: 11 (100%)	Yes: 12 (100%)	Yes: 11 (100%)
	Trunk curl	Absence: 11 (100%)	Absence: 12 (100%)	Absence: 11 (100%)
	Limb grasping	Present: 11 (100%)	Present: 12 (100%)	Present: 11 (100%)
	Visual Placing	Before vibrasse contact: 11 (100%)	Before vibrasse contact: 12 (100%)	Before vibrasse contact: 11 (100%)
	Grip Strength	Moderate grip, effective: 11 (100%)	Moderate grip, effective: 12 (100%)	Moderate grip, effective: 11 (100%)
	Body Tone	Slight resistance: 11 (100%)	Slight resistance: 12 (100%)	Slight resistance: 11 (100%)
	Head morphology	Normal: 11 (100%)	Normal: 12 (100%)	Normal: 11 (100%)
	Corneal Reflex	Active single eye blink: 11 (100%)	Active single eye blink: 12 (100%)	Active single eye blink: 11 (100%)
section 4	**Supinate restraint**			
	Lacrimation	None: 11 (100%)	None: 12 (100%)	None: 11 (100%)
	Whisker morphology	Normal: 11 (100%)	Normal: 12 (100%)	Normal: 11 (100%)
	Tooth morphology	Normal: 11 (100%)	Normal: 12 (100%)	Normal: 11 (100%)
	Provoked Biting	Present: 11 (100%)	Present: 12 (100%)	Present: 11 (100%)
	Salivation	None: 11 (100%)	None: 12 (100%)	None: 11 (100%)
	Heart Rate	Normal: 11 (100%)	Normal: 12 (100%)	Normal: 11 (100%)
	Abdominal tone	Slight resistance: 11 (100%)	Slight resistance: 12 (100%)	Slight resistance: 11 (100%)
	Skin color	Pink: 11 (100%)	Pink: 12 (100%)	Pink: 11 (100%)
	Toe pinch	Slight withdrawal, not brisk: 0 (0%)	Slight withdrawal, not brisk: 1 (8%)	Slight withdrawal, not brisk: 0 (0%)
		moderate withdrawal, not brisk: 11 (100%)	moderate withdrawal, not brisk: 11 (92%)	moderate withdrawal, not brisk: 11 (100%)

Appendix Table S1 The modified SHIRPA primary screen in WT, HTT_{SD} and HTT_{SA} mice.

Results are presented in percentages unless otherwise indicated. No significant differences between genotypes were observed.

Appendix Table S2 : Statistical information for main figures

Figure 1B	Unpaired t test	P value	Label
anterograde velocity	WT siMecp2 vs WT siCtl	0.0001	***
retrograde velocity	WT siMecp2 vs WT siCtl	0.002	**
mean speed	WT siMecp2 vs WT siCtl	0.0004	***
Flow	WT siMecp2 vs WT siCtl	0.023	*

Figure 1C	Kruskal-Wallis test	P value	Label
Cell viability		0.1418	ns

Figure 1D	One way ANOVA	P value	Label
mean velocity		0.0002	***
	Tukey's multiple comparisons test		Label
	WT vs. HTTSD		***
	WT vs. HTTSA		ns
	HTTSD vs. HTTSA		ns
anterograde velocity		< 0,0001	****
	Tukey's multiple comparisons test		Label
	WT vs. HTTSD		****
	WT vs. HTTSA		ns
	HTTSD vs. HTTSA		****
Retrograde velocity		0.0007	***
	Tukey's multiple comparisons test		Label
	WT vs. HTTSD		ns
	WT vs. HTTSA		**
	HTTSD vs. HTTSA		**
Flow		0.1383	ns

Figure 1E	One way ANOVA	P value	Label
		< 0,0001	****
	Tukey's multiple comparisons test		Label
mean velocity	WT siCtl vs. WT siMecp2		**
	WT siCtl vs. SD siMecp2		ns
	WT siCtl vs. SA siMecp2		*
	WT siMecp2 vs. SD siMecp2		****
	WT siMecp2 vs. SA siMecp2		ns
	DD siMecp2 vs. SA siMecp2		****

		< 0,0001	****
	Tukey's multiple comparisons test		Label
anterograde velocity	WT siCtl vs. WT siMecp2		****
	WT siCtl vs. SD siMecp2		ns
	WT siCtl vs. SA siMecp2		****
	WT siMecp2 vs. SD siMecp2		***
	WT siMecp2 vs. SA siMecp2		ns
	DD siMecp2 vs. SA siMecp2		****
		0.0043	**
	Tukey's multiple comparisons test		Label
Retrograde velocity	WT siCtl vs. WT siMecp2		*
	WT siCtl vs. SD siMecp2		ns
	WT siCtl vs. SA siMecp2		ns
	WT siMecp2 vs. SD siMecp2		*
	WT siMecp2 vs. SA siMecp2		**
	DD siMecp2 vs. SA siMecp2		ns
		0.0131	*
	Tukey's multiple comparisons test		Label
Flow	WT siCtl vs. WT siMecp2		*
	WT siCtl vs. SD siMecp2		ns
	WT siCtl vs. SA siMecp2		ns
	WT siMecp2 vs. SD siMecp2		*
	WT siMecp2 vs. SA siMecp2		ns
	DD siMecp2 vs. SA siMecp2		ns

Figure 1F	Kruskal-Wallis test	P value	Label
Cell viability		0.157	ns

Figure 2A	Mann-Whitney	P value	Label
BDNF content ratio	WT vs KO	0,0406	*
	WT vs KO HTTSD	0.9988	ns
	WT vs KO HTTSA	0,0326	*
	KO vs KO HTTSD	0,0333	*
	KO vs KO HTTSA	0.8853	ns
	KO HTTSD vs KO HTTSA	0,0317	*

Figure 2A	Mann-Whitney	P value	Label

PSD95	WT vs KO	0.0021	**
	WT vs KO HTTSD	0.9906	ns
	WT vs KO HTTSA	0.0365	*
	KO vs KO HTTSD	0.0041	**
	KO vs KO HTTSA	0.5072	ns
	KO HTTSD vs KO HTTSA	0.0676	ns

Figure 2A	Mann-Whitney	P value	Label
TrkBp	WT vs KO	0.0532	ns
	WT vs KO HTTSD	0.9984	ns
	WT vs KO HTTSA	0.0557	ns
	KO vs KO HTTSD	0.0366	*
	KO vs KO HTTSA	>0.9999	ns
	KO HTTSD vs KO HTTSA	0.0385	*

Figure 2B	Log-rank (Mantel-Cox) Test	P value	Label
Survival curves	KO vs KO HTTSD	0.0264	*
	KO HTTSD vs KO HTTSA	0.0065	**

Figure 2C	One way ANOVA	P value	Label
P30		0.0004	***
	Tukey's multiple comparisons test		Label
	KO HTTSD vs KO	0.2683	ns
	KO HTTSD vs KO HTTSA	0.2845	ns
	KO HTTSD vs WT	0.0278	*
	KO HTTSA vs KO	>0.9999	ns
	KO vs WT	0.0007	***
	KO HTTSA vs WT	0.0008	***
P50		<0.0001	****
	Tukey's multiple comparisons test		Label
	KO HTTSD vs KO	0.0216	*
	KO HTTSD vs KO HTTSA	0.1101	ns
	KO HTTSD vs WT	<0.0001	****
	KO HTTSA vs KO	0.9697	ns
	KO vs WT	<0.0001	****
	KO HTTSA vs WT	<0.0001	****

Figure 2D	Kruskal-Wallis test	P value	Label
P35		0.0027	**
	Dunn's multiple comparisons test		Label
	KO vs WT	0.0047	**
	KO HTTSD vs WT	>0.9999	ns

	KO HTTSA vs WT	0.4928	ns
	KO HTTSD vs KO	0.0099	**
	KO HTTSA vs KO	0.3212	ns
	KO HTTSD vs KO HTTSA	>0.9999	ns
P55		<0.0001	****
	Dunn's multiple comparisons test		Label
	KO vs WT	<0.0001	****
	KO HTTSD vs WT	0.5789	ns
	KO HTTSA vs WT	0.0001	***
	KO HTTSD vs KO	0.0215	*
	KO HTTSA vs KO	>0.9999	ns
	KO HTTSD vs KO HTTSA	0.0229	*

Figure 2E	Kruskal-Wallis test	P value	Label
P35		0.0042	**
	Dunn's multiple comparisons test		Label
	KO vs WT	0.0291	*
	KO HTTSD vs WT	0.5387	ns
	KO HTTSA vs WT	0.0012	**
	KO HTTSD vs KO	0.0961	ns
	KO HTTSA vs KO	0.2148	ns
	KO HTTSD vs KO HTTSA	0.0049	**
P55		0.0003	***
	Fisher's LSD		Label
	KO vs WT	<0.0001	****
	KO HTTSD vs WT	0.0776	ns
	KO HTTSA vs WT	0.0008	***
	KO HTTSD vs KO	0.0133	*
	KO HTTSA vs KO	0.6669	ns
	KO HTTSD vs KO HTTSA	0.055	ns

Figure 2F	Kruskal-Wallis test	P value	Label
P35		0.0028	**
	Dunn's multiple comparisons test		Label
	WT vs. KO SD	0.1862	ns
	WT vs. KO	0.0174	*
	WT vs. KO SA	0.0024	**
	KO SD vs. KO	>0.9999	ns
	KO SD vs. KO SA	>0.9999	ns

	KO vs. KO SA	>0.9999	ns

Figure 3A	Mann-Whitney	P value	Label
Vehicle vs FK506	SiCtl	0.0188	*
	Si Mecp2	0.0411	*

Figure 3C	One way ANOVA	P value	Label
		< 0,0001	****
	Tukey's multiple comparisons test		Label
mean speed	siCtl Vehicle vs. siCtl FK506		****
	siCtl Vehicle vs. siMecp2 Vehicle		****
	siCtl Vehicle vs. siMecp2 FK506		Ns
	siCtl FK506 vs. siMecp2 Vehicle		****
	siCtl FK506 vs. siMecp2 FK506		**
	siMecp2 Vehicle vs. siMecp2 FK506		****
		< 0,0001	****
	Tukey's multiple comparisons test		Label
anterograde velocity	siCtl Vehicle vs. siCtl FK506		*
	siCtl Vehicle vs. siMecp2 Vehicle		****
	siCtl Vehicle vs. siMecp2 FK506		ns
	siCtl FK506 vs. siMecp2 Vehicle		****
	siCtl FK506 vs. siMecp2 FK506		ns
	siMecp2 Vehicle vs. siMecp2 FK506		****
		< 0,0001	****
	Tukey's multiple comparisons test		Label
Retrograde velocity	siCtl Vehicle vs. siCtl FK506		ns
	siCtl Vehicle vs. siMecp2 Vehicle		***
	siCtl Vehicle vs. siMecp2 FK506		ns
	siCtl FK506 vs. siMecp2 Vehicle		****
	siCtl FK506 vs. siMecp2 FK506		**
	siMecp2 Vehicle vs. siMecp2 FK506		**
		< 0,0001	****
	Tukey's multiple comparisons test		Label
Flow	WT siCtl vs. WT siMecp2		**
	WT siCtl vs. SD siMecp2		***
	WT siCtl vs. SA siMecp2		ns
	WT siMecp2 vs. SD siMecp2		****
	WT siMecp2 vs. SA siMecp2		***
	DD siMecp2 vs. SA siMecp2		****
	Tukey's multiple comparisons test		Label
Nb vesicles	WT siCtl vs. WT siMecp2		ns
	WT siCtl vs. SD siMecp2		ns
	WT siCtl vs. SA siMecp2		ns
	WT siMecp2 vs. SD siMecp2		*

	WT siMecp2 vs. SA siMecp2		ns
	SD siMecp2 vs. SA siMecp2		ns

Figure 4A	Mann-Whitney	P value	Label
pS421 level	KO veh vs KO FK506	0.0159	*

Figure 4B	Log-rank (Mantel-Cox) Test	P value	Label
Survival curves	KO veh vs KO FK506	0.0201	*
	KO HTTSA FK506 vs KO FK506	0.0019	**

Figure 4C	Mann-Whitney	P value	Label
P55	KO veh vs KO FK506	0.0451	*

Figure 4D	Mann-Whitney	P value	Label
P35	KO HTTSA FK506 vs KO FK506	0.0121	*
	KO veh vs KO FK506	0.0339	*
P55	KO HTTSA FK506 vs KO FK506	0.0193	*

Figure 4E	Mann-Whitney	P value	Label
P50	KO veh vs KO FK506	0.0002	***
	KO HTTSA FK506 vs KO FK506	0.0498	*

Figure 4F	Mann-Whitney	P value	Label
P60	KO veh vs KO FK506	0.0498	*
	KO HTTSA FK506 vs KO FK506	0.0006	***

Appendix Table S3 : Statistical information for supplementary figures

Figure EV1B	Unpaired t test	P value	Label
moving vesic nb /axon	WT siMecp2 vs WT siCtl	0.7797	ns

Figure EV1C	Kruskal-Wallis test	P value	Label
		0.9929	ns

Figure EV1D	One way ANOVA	P value	Label
		0.7752	ns
	Tukey's multiple comparisons test		Label
mean speed	WT vs. HTTSD		ns
	WT vs. HTTSA		ns
	HTTSD vs. HTTSA		ns

Figure EV1F	One way ANOVA	P value	Label
		0.838	ns
	Tukey's multiple comparisons test		Label
mean speed	WT siCtl vs. WT siMecp2		ns
	WT siCtl vs. SD siMecp2		ns
	WT siCtl vs. SA siMecp2		ns
	WT siMecp2 vs. SD siMecp2		ns
	WT siMecp2 vs. SA siMecp2		ns
	SD siMecp2 vs. SA siMecp2		ns

Figure EV2F	Kruskal-Wallis test	P value	Label
P55		0.0015	**
	Dunn's multiple comparisons test		Label
	KO vs WT	0.0085	**
	KO HTTSD vs WT	0.053	ns
	KO HTTSA vs WT	0.0039	**
	KO HTTSD vs KO	>0.9999	ns
	KO HTTSA vs KO	>0.9999	ns
	KO HTTSD vs KO HTTSA	>0.9999	ns

Figure EV2H	Kruskal-Wallis test	P value	Label
8 p.m		<0.0001	****
	Dunn's multiple comparisons test		Label
	KO vs WT	0.0003	***
	KO HTTSD vs WT	0.4609	ns
	KO HTTSA vs WT	<0.0001	****
	KO HTTSD vs KO	0.3594	ns
	KO HTTSA vs KO	>0.9999	ns

	KO HTTSD vs KO HTTSA	0.0325	*

Figure EV3A	Unpaired t test	P value	Label
pS421 level	KO veh vs KO FK506	0.0234	*

Figure EV3C	Mann-Whitney	P value	Label
pS421 level	KO veh vs KO FK506	0.2	ns
