# Supplementary material for: Huntingtin phosphorylation governs BDNF homeostasis and improves the phenotype of Mecp2 knockout mice
Source: EMBO Mol Med. 2020 Jan 8;12(2):e10889. doi: 10.15252/emmm.201910889 (PMC7005633; doi:10.15252/emmm.201910889)
Supplement: Supplementary file 2 — Expanded View Figures PDF [file EMMM-12-e10889-s002.pdf]

Expanded View Figures

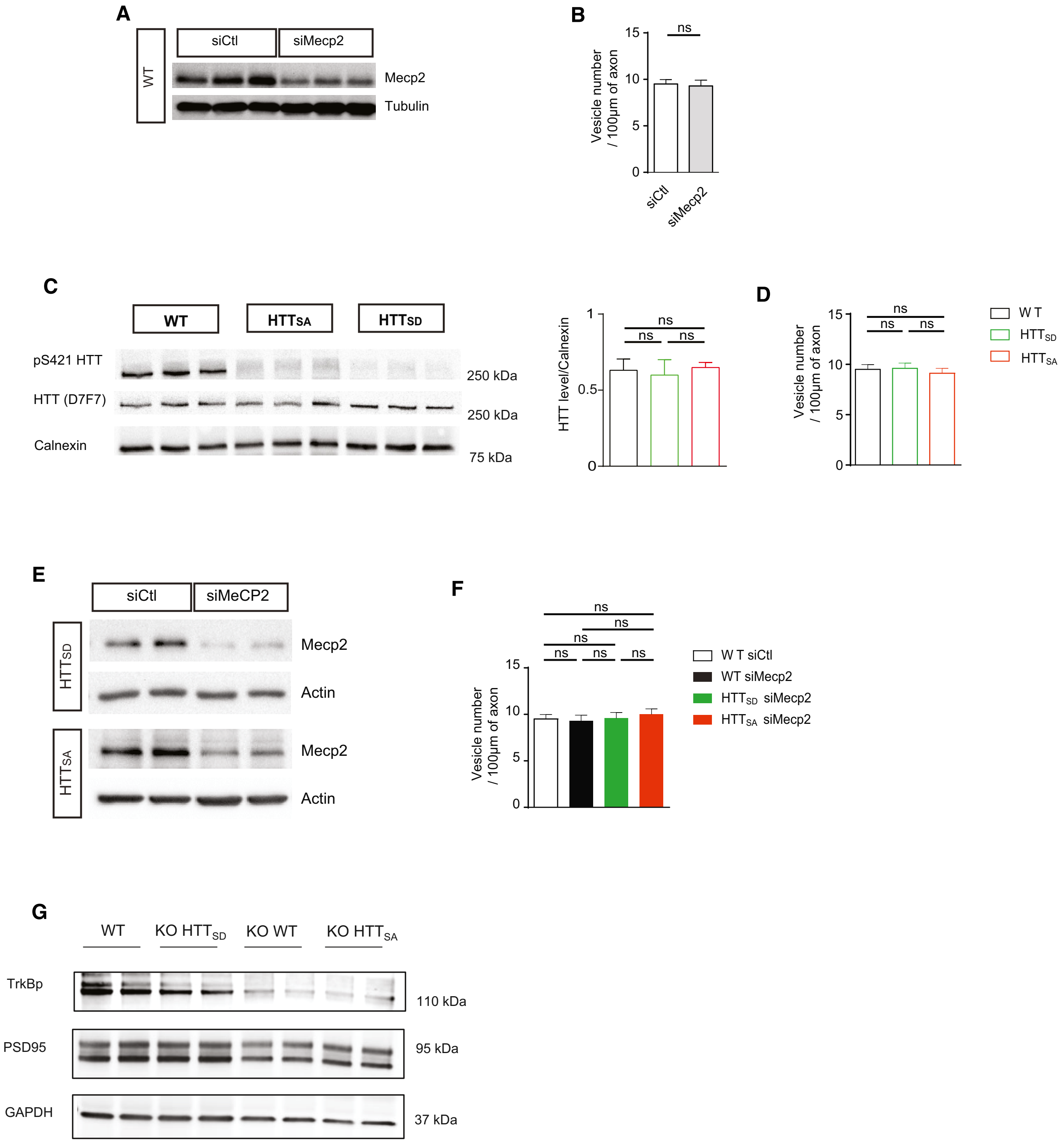

Figure EV1.

© 2020 The Authors

Figure EV1. Mecp2 levels and axonal vesicle number in cortical *Mecp2*-silenced neurons.

A Western blot analysis of DIV 5 cortical neurons transfected with siMecp2 or siControl (siCtl) to assess Mecp2 protein levels.

B We quantified the number of BDNF-mCherry-containing vesicles trafficking within cortical axons transfected with siMecp2 or siControl (siCtl). Data are presented as the mean ± SEM of at least three independent experiments, with 94 siCtl and 81 siMecp2 axons and 894 siCtl and 753 siMecp2 vesicles (unpaired *t*-test).

C Brain extracts from WT, WT HTT_{SD}, and WT HTT_{SA} mice were analyzed by Western blotting for total HTT and phospho-HTT. Quantification of $n = 3$ samples per genotype did not reveal any significant differences on total HTT. The mutation of HTT S421 into alanine or aspartic acid abrogated the recognition of phospho-HTT by the phospho-specific antibody, further demonstrating the specificity of HTT phosphorylation at S421. Data are presented as means ± SEM, and one-way ANOVA test followed by Tukey's post-hoc analysis for multiple comparisons.

D We quantified the number of BDNF-mCherry-containing vesicles trafficking within WT, HTT_{SA}, or HTT_{SD} cortical axons. Data are presented as the mean ± SEM of at least three independent experiments, with 80 axons and 700 vesicles per condition (one-way ANOVA test followed by Tukey's post-hoc analysis for multiple comparisons).

E Western blot analysis of DIV 5 HTT_{SA} or HTT_{SD} cortical neurons transfected with siMecp2 or siControl (siCtl) to assess Mecp2 protein levels.

F Quantification of the number of BDNF-mCherry-containing vesicles trafficking within WT, HTT_{SA}, or HTT_{SD} cortical axons transfected with siMecp2 or siControl (siCtl). Data are presented as the mean ± SEM of at least three independent experiments, with WT (n = 753 vesicles/81 axons), HTT_{SA} (n = 812 vesicles/81 axons), or HTT_{SD} (n = 787 vesicles/82 axons) (one-way ANOVA test followed by Tukey's post-hoc analysis for multiple comparisons). ns = not significant.

G Representative Western blot analysis of PSD-95 and phospho-TrkB protein levels in striatum samples from WT, KO HTT_{SD}, KO WT, and KO HTT_{SA} mice.

Source data are available online for this figure.

Figure EV2. Phenotypic characterization of WT, HTT_{SD}, HTT_{SA} (A, B, C, and D) mice and WT, KO/HTT_{SD}, KO/HTT_{SA} (E, F, G, and H) mice.

A There were no significant differences in body weight between 6-month-old WT, HTT_{SD}, and HTT_{SA} mice (WT: n = 11; HTT_{SD}: n = 6, and HTT_{SA}: n = 10).

B No significant differences in the distance travelled in the open-field between 4-month-old WT, HTTSD, and HTTSA mice (WT: n = 10; HTT_{SD}: n = 6 HTT_{SA}: n = 11).

C No differences between genotypes in the accelerating Rotarod test for assessing motor coordination of 6-month-old WT (n = 11), HTT_{SD} (n = 11), and HTT_{SA} mice (n = 12).

D No differences in forelimb strength of 6-month-old WT (n = 11), HTT_{SD} (n = 11), and HTT_{SA} mice (n = 12) as assessed by the grip strength test.

E No significant differences in body weight between KO/HTT_{SD} (n = 32), KO/HTT_{SA} (n = 24), and KO mice (n = 24) at different postnatal timepoints.

F Distance travelled during the open-field test by WT (n = 17), KO (n = 20), KO/HTT_{SD} (n = 19), and KO/HTT_{SA} mice (n = 17) at P35 and P55. Results are expressed in meters.

G, H Results of 24-h Phenorack monitoring to measure the spontaneous activity of 12 WT, 12 KO, 8 KO/HTT_{SD}, and 7 KO/HTT_{SA} mice at P45 (G). Distance travelled at 8 AM versus 8 PM (H).

Data information: Kruskal–Wallis test with Dunn's comparison; *$P < 0.05$, **$P < 0.01$, ***$P < 0.001$, ns = not significant. Data are presented as the means ± SEM.

© 2020 The Authors

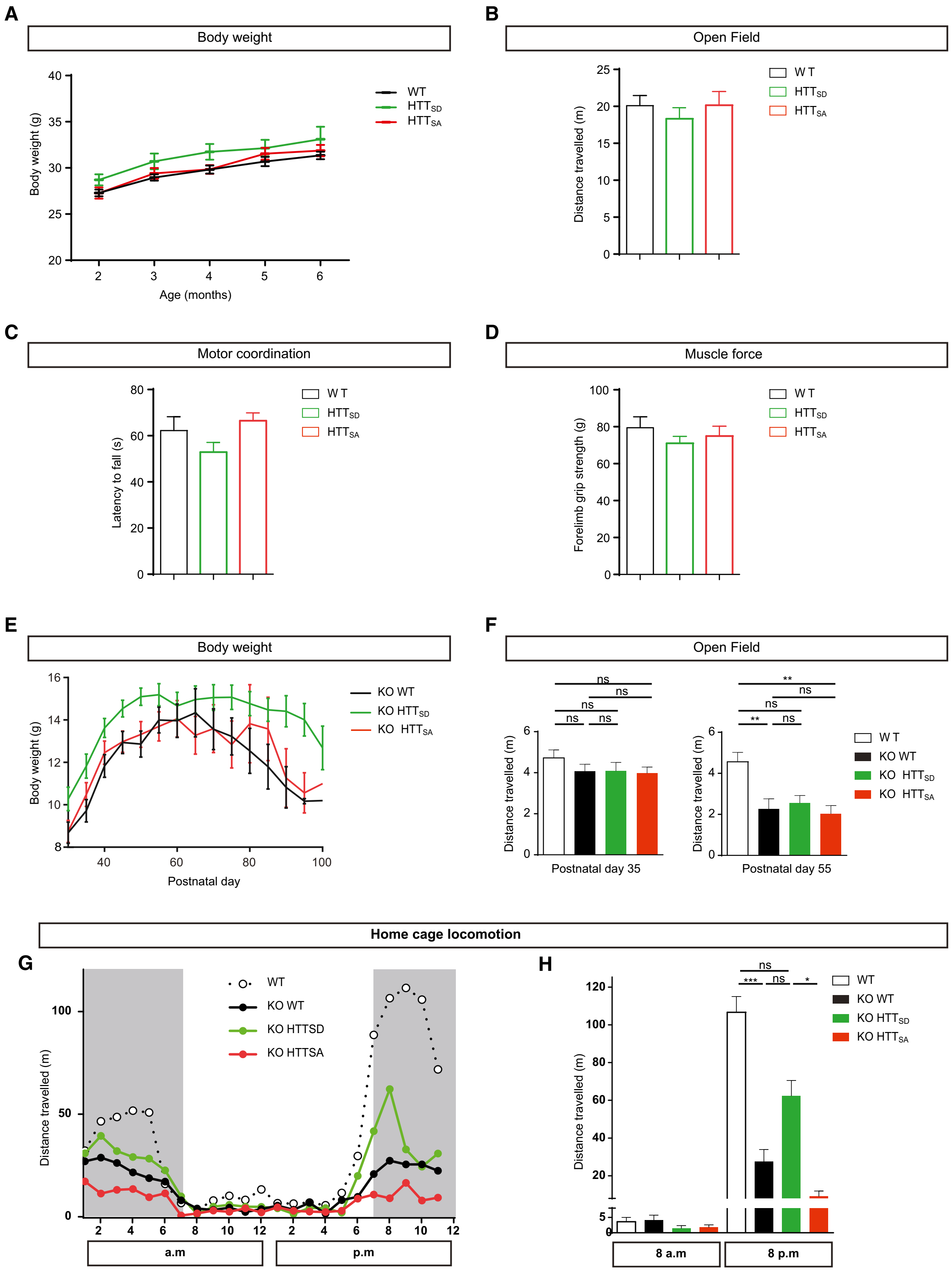

Figure EV2.

 © 2020 The Authors

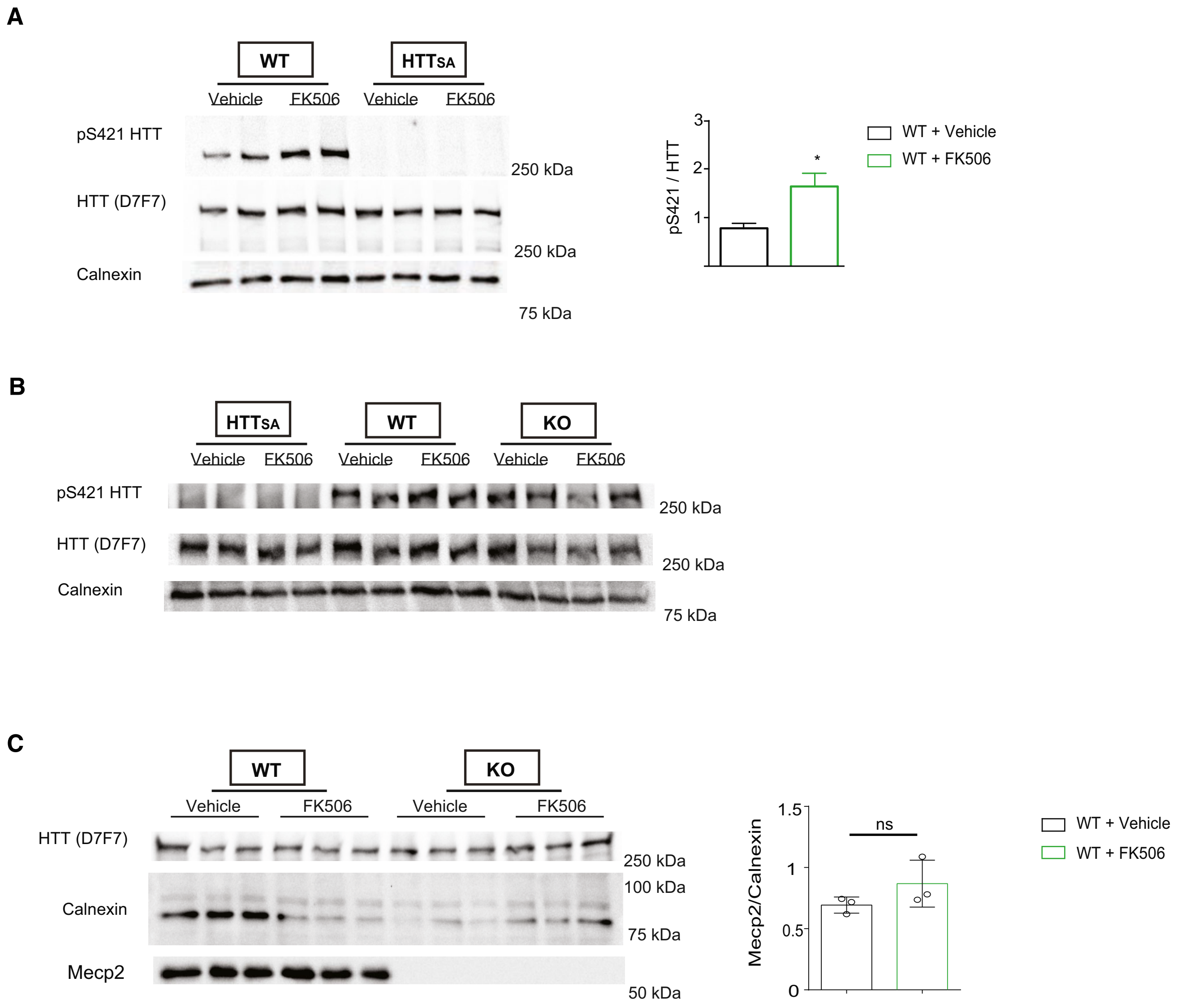

Figure EV3. Acute and chronic FK506 treatment of WT, HTT$_{SA}$, and *Mecp2* KO mice.

A Western blot of HTT S421 phosphorylation. We treated WT and HTT$_{SA}$ 30-day-old mice intraperitoneally with FK506 (5 mg/kg) or vehicle and analyzed brain extracts for endogenous HTT phosphorylation by Western blotting, 2 h after administration (WT FK506 = 3, WT Vehicle = 6, HTT$_{SA}$ FK506 = 3, HTT$_{SA}$ Vehicle = 3). The relative protein level of phospho-HTT was normalized on total HTT protein level and is presented as the ratio. Data are presented as the means ± SEM, *$P < 0.05$, Mann–Whitney test.

B Western blot of HTT S421 phosphorylation. We treated WT, HTT$_{SA}$, and *Mecp2* KO 30-day-old mice intraperitoneally with FK506 (5 mg/kg) or vehicle chronically for 20 days and analyzed brain extracts for endogenous HTT phosphorylation by Western blotting, 2 h after the last administration (KO FK506 n = 2, KO Vehicle n = 2, WT FK506 = 2, WT Vehicle = 2, HTT$_{SA}$ FK506 = 2, HTT$_{SA}$ Vehicle = 2).

C Western blot of HTT and Mecp2. We treated WT and *Mecp2* KO 30-day-old mice intraperitoneally with FK506 (5 mg/kg) or vehicle chronically for 20 days (WT FK506 = 3, WT Vehicle = 3, HTT$_{SA}$ FK506 = 3, HTT$_{SA}$ Vehicle = 3). Data are presented as the means ± SEM, ns = not significant, Mann–Whitney test.

Source data are available online for this figure.

Figure EV4. Absence of cytotoxicity and increased neuronal activity after chronic FK506 treatment.

A FK506 did not increase the levels of cleaved caspase-3, as shown by immunostaining. Representative images of Striatum labeled with Casp3 (green) and DAPI of 50-day-old *Mecp2* KO mice treated with FK506 (right panel) or vehicle (left panel) for 20 days.

B Absence of apoptosis in the dorsal striatum of *Mecp2*-knockout mice treated chronically with FK505 or with Vehicle. TUNEL assay was used to detect potential apoptotic neurons in 50-day-old *Mecp2*-deficient mice (n = 3 for each stage). Left panel: detection of cells containing fragmented DNA after desoxyribonuclease I treatment of a representative dorsal striatum section originating from one *Mecp2*-knockout and one wild-type animal. No labeled cells were visible after counting all striatum sections of 50-day-old *Mecp2*-knockout mice.

C FK506 tends to increase the number of cFos-positive neurons. Representative images of striatum labeled with cFos (green) of 50-day-old KO mice treated with FK506 (right panel) or vehicle (middle panel) for 20 days.

D Vertical scatter plot representing the number of cFos positive neurons counted in the dorsal striatum of P50 Wt placebo (n = 2)-, KO placebo (n = 3)-, and KO FK506 (n = 3)-treated mice in a picture of length 650 μm and width 445 μm. Each dot represents the mean of three successive sections.

© 2020 The Authors

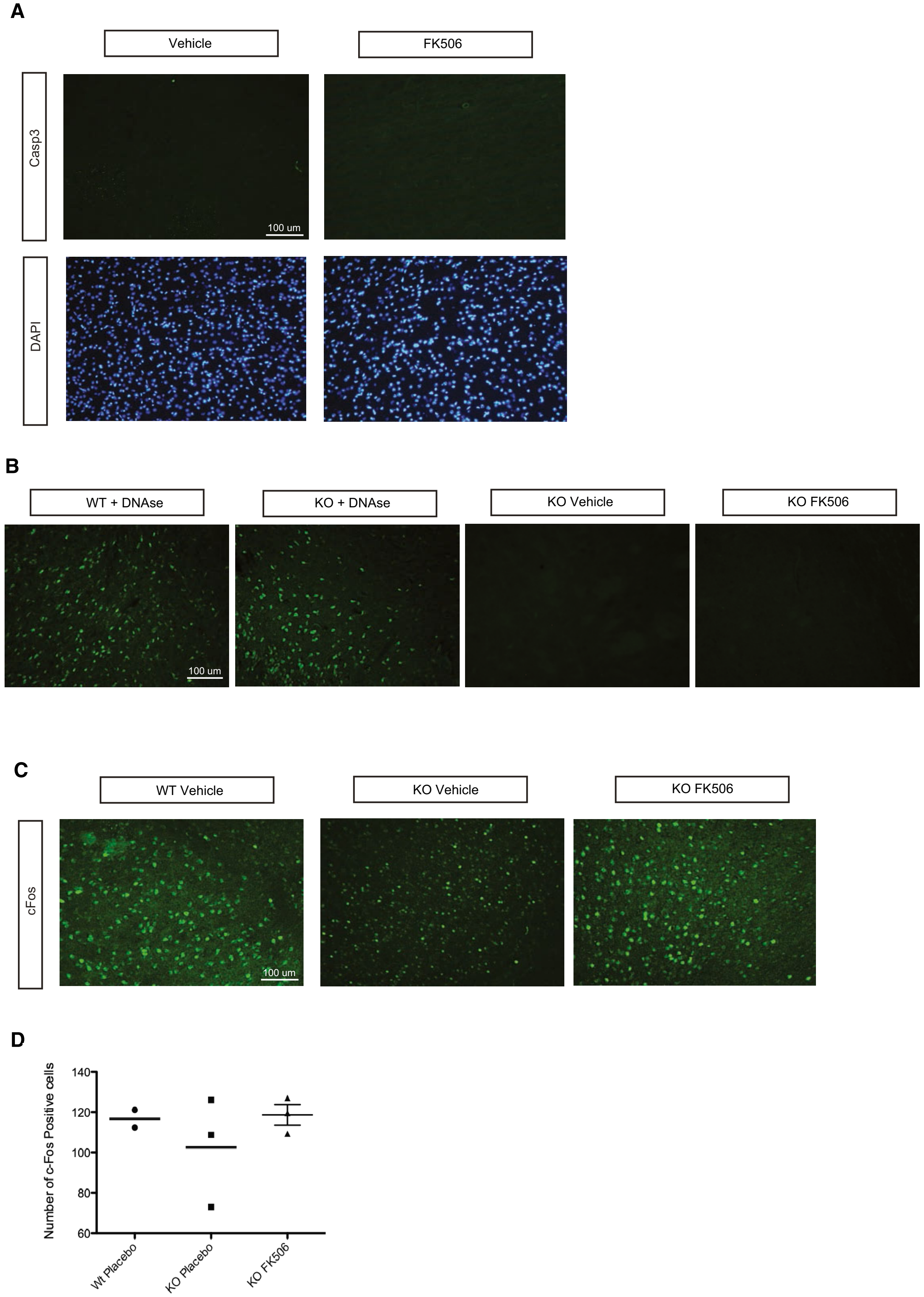
A
Vehicle
FK506
Casp3
DAPI
100 um
B
WT + DNAse
KO + DNAse
KO Vehicle
KO FK506
100 um
C
WT Vehicle
KO Vehicle
KO FK506
cFos
100 um
D
Number of c-Fos Positive cells
140
120
100
80
60
Wt Placebo
KO Placebo
KO FK506

Figure EV4.

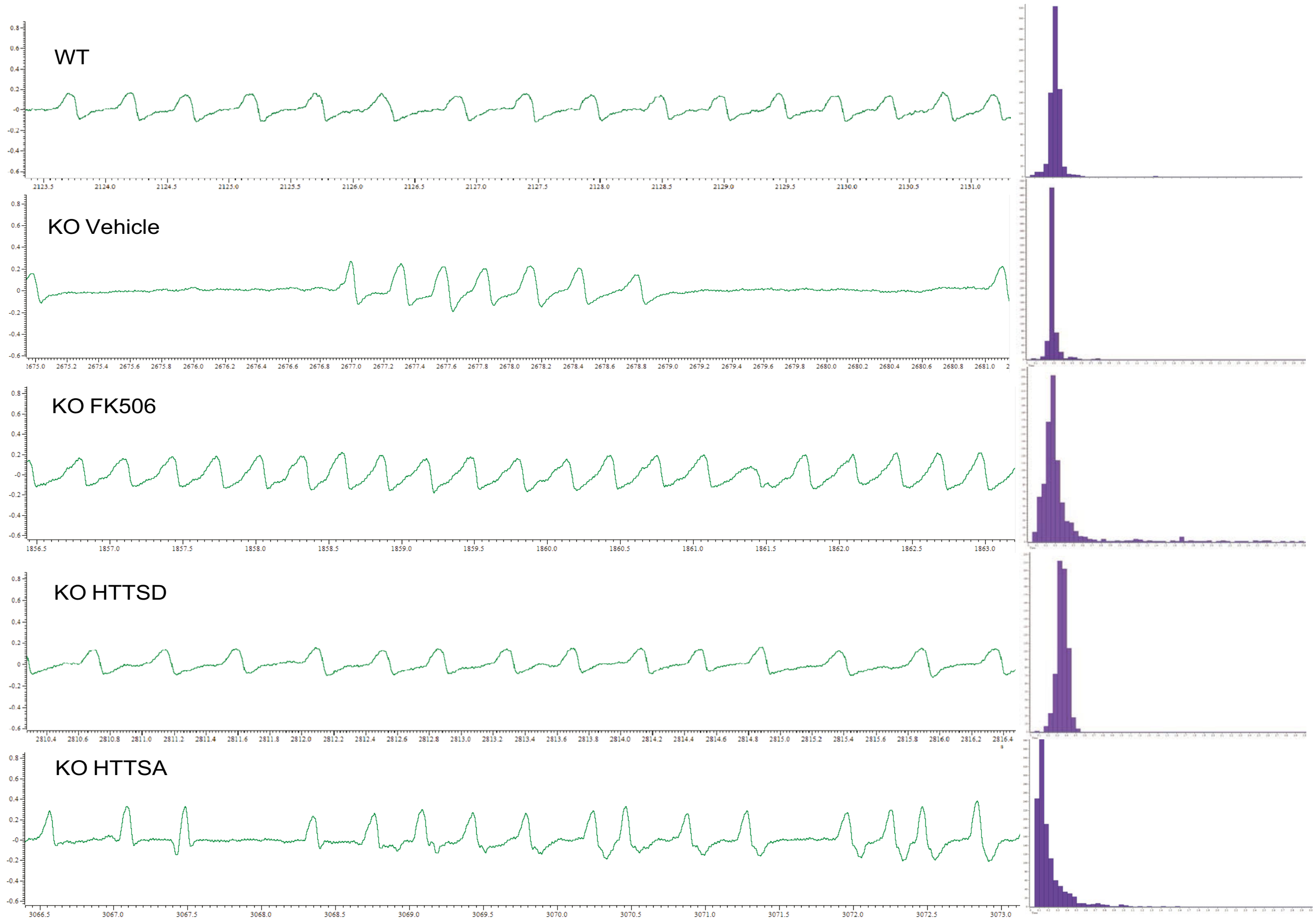

Figure EV5. Breathing patterns in 55-day-old WT, KO Vehicle, KO FK506, KO/HTT_{SD}, and KO/HTT_{SA} mice.

Left: Typical plethysmographic recordings of breathing (inspiration upward) performed in quiet, 55-day-old WT, KO Vehicle, KO FK506, KO/HTT_{SD}, and KO/HTT_{SA} mice. Right: Distribution of frequency values recorded in WT, KO Vehicle, KO FK506, KO/HTT_{SD}, and KO/HTT_{SA} mice at P55. Frequency histograms represent the number of occurrences (ordinate) of breathing frequency during 4 consecutive minutes separated in 0.05-s time windows.

 © 2020 The Authors
