# Supplementary material for: Huntingtin phosphorylation governs BDNF homeostasis and improves the phenotype of Mecp2 knockout mice
Source: EMBO Mol Med. 2020 Jan 8;12(2):e10889. doi: 10.15252/emmm.201910889 (PMC7005633; doi:10.15252/emmm.201910889)
Supplement: Supplementary file 3 — Movie EV1 [file EMMM-12-e10889-s003.zip › Movie_EV1/Movie EV1.rtf]

Movie EV1 BDNF vesicle trafficking in axons of cortical Mecp2-silenced neurons.BDNF-mCherry-containing vesicles trafficking within WT cortical axon transfected with siControl (siCtl) or siMecp2. HTTSD rescues BDNF-mCherry trafficking in siMecp2 cortical axons.
