# Supplementary material for: Huntingtin phosphorylation governs BDNF homeostasis and improves the phenotype of Mecp2 knockout mice
Source: EMBO Mol Med. 2020 Jan 8;12(2):e10889. doi: 10.15252/emmm.201910889 (PMC7005633; doi:10.15252/emmm.201910889)
Supplement: Supplementary file 4 — Movie EV2 [file EMMM-12-e10889-s004.zip › Movie_EV2/Movie EV2.rtf]

Movie EV2 BDNF vesicle trafficking in axons of cortical Mecp2-silenced neurons treated with FK506.BDNF-mCherry-containing vesicles trafficking within cortical axons transfected with siMecp2 or siControl (siCtl) and treated with 1 ƒÊM FK506 or vehicle for 1 h. FK506 rescues BDNF-mCherry trafficking in siMecp2 cortical axons.
