# Supplementary material for: Huntingtin phosphorylation governs BDNF homeostasis and improves the phenotype of Mecp2 knockout mice
Source: EMBO Mol Med. 2020 Jan 8;12(2):e10889. doi: 10.15252/emmm.201910889 (PMC7005633; doi:10.15252/emmm.201910889)

Full unedited gel for Figure EV1A

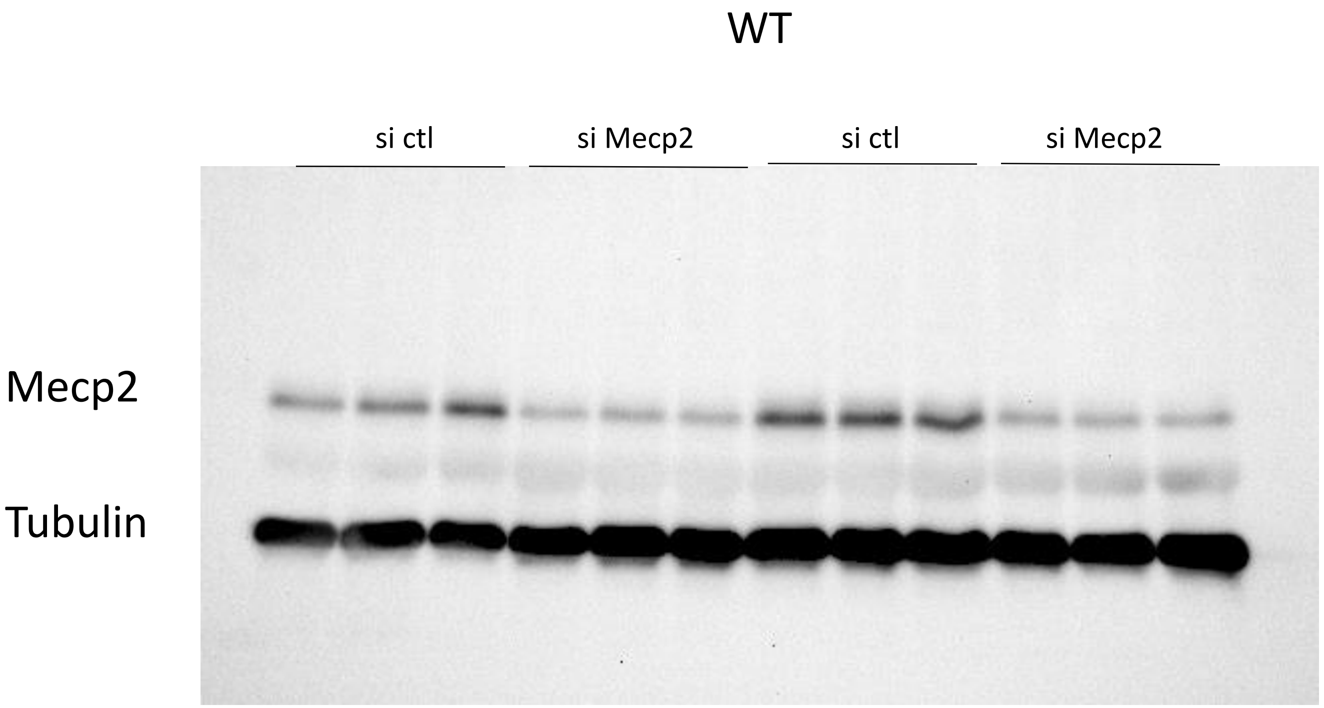

Full unedited gel for Figure EV1C

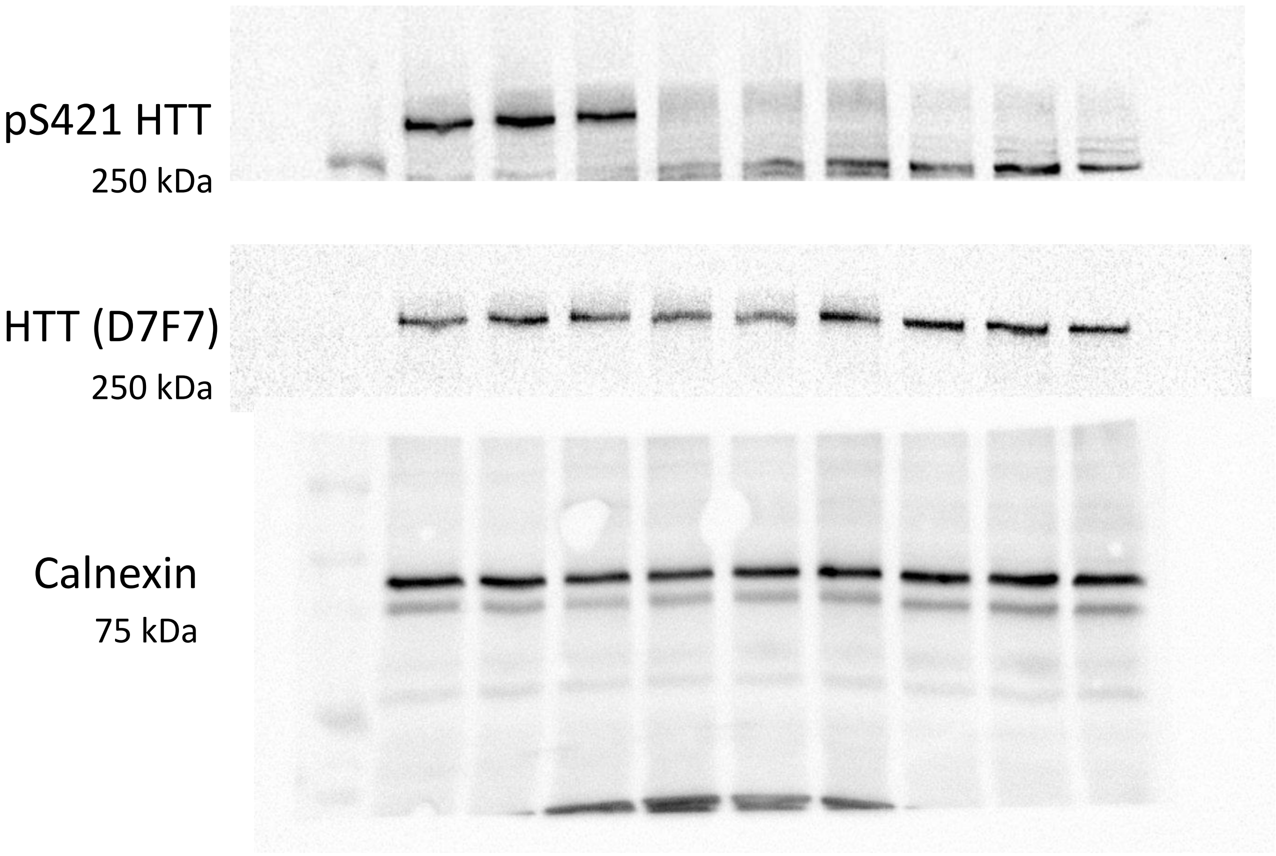

Full unedited gel for Figure EV1E

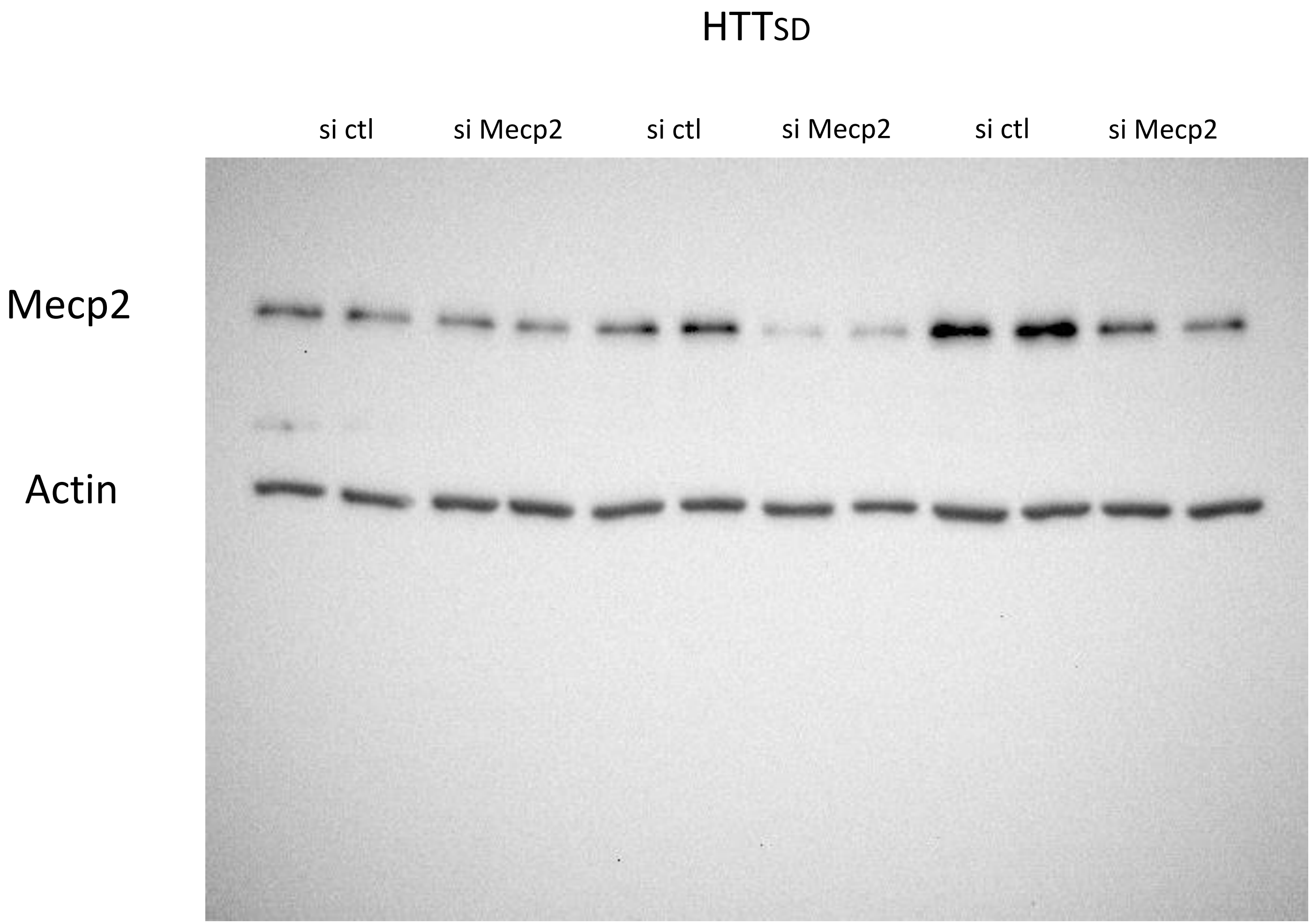

Full unedited gel for Figure EV1E

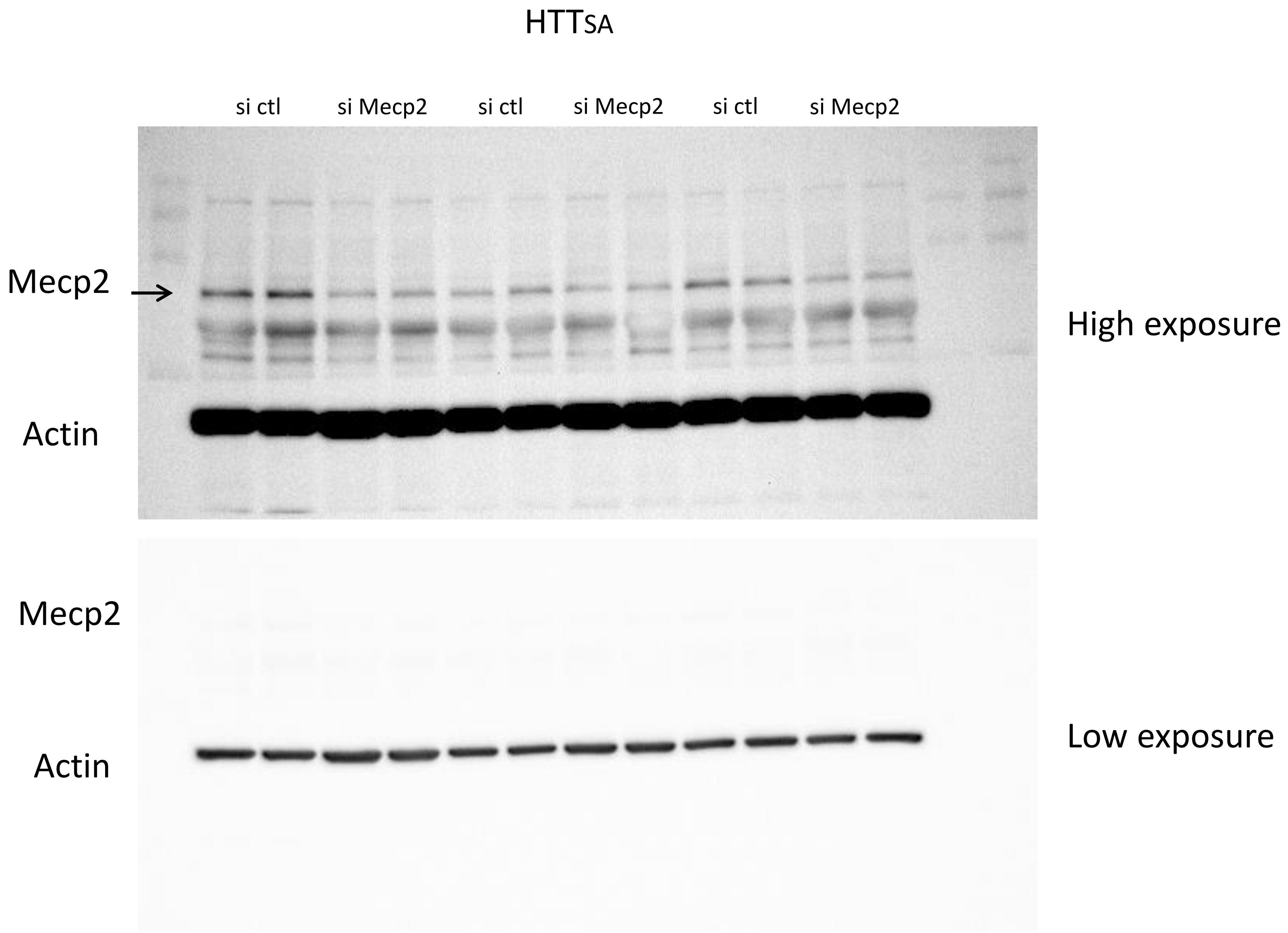

Full unedited gel for Figure EV1G

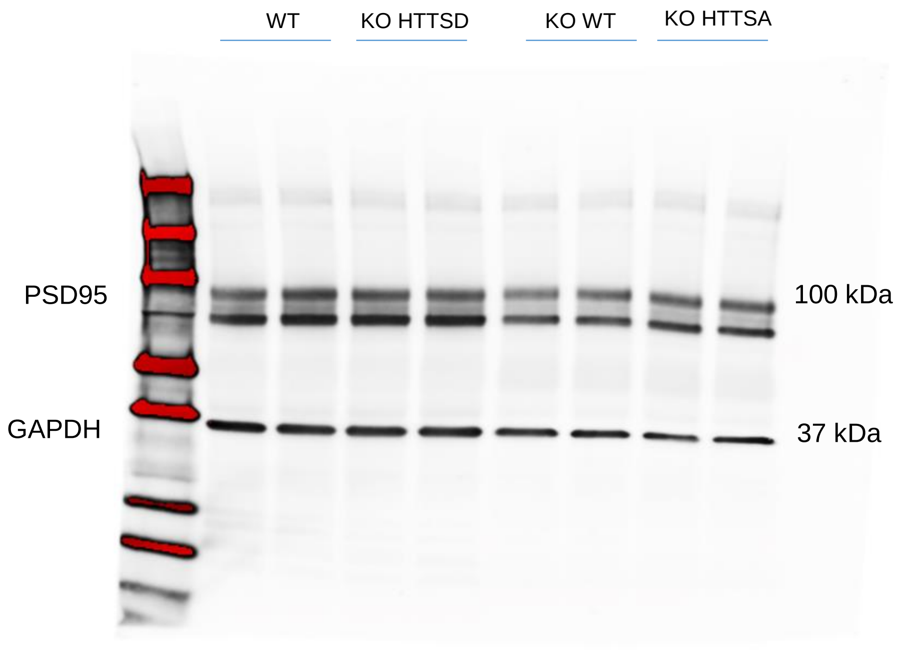

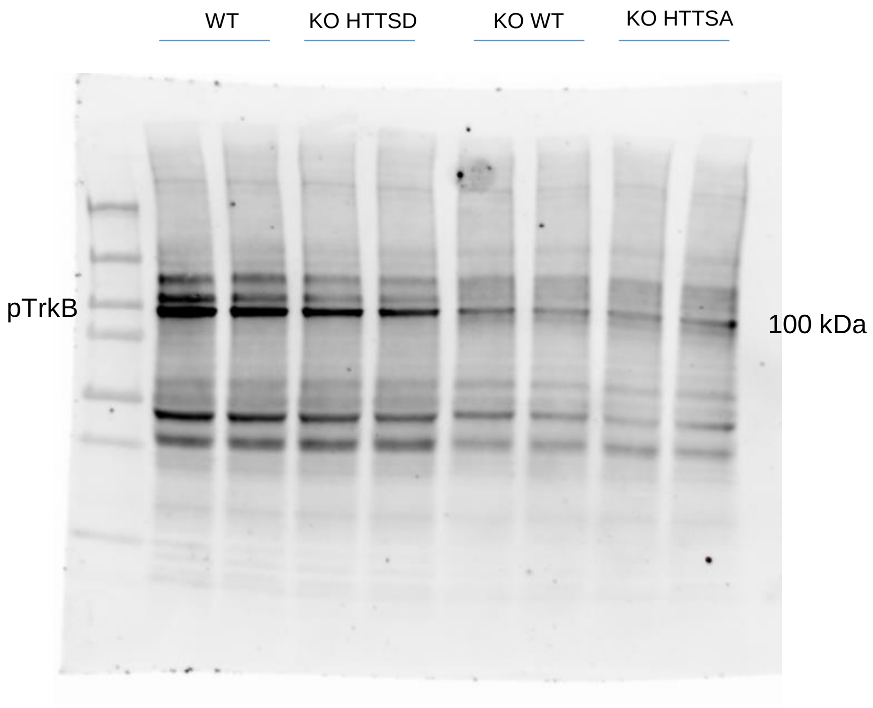

Supplement: Supplementary file 5 — Source Data for Expanded View [file EMMM-12-e10889-s007.zip › EV_source_data/Figure_EV1_source_data.pdf]
