# Supplementary material for: Huntingtin phosphorylation governs BDNF homeostasis and improves the phenotype of Mecp2 knockout mice
Source: EMBO Mol Med. 2020 Jan 8;12(2):e10889. doi: 10.15252/emmm.201910889 (PMC7005633; doi:10.15252/emmm.201910889)

Full unedited gel for Figure EV3A

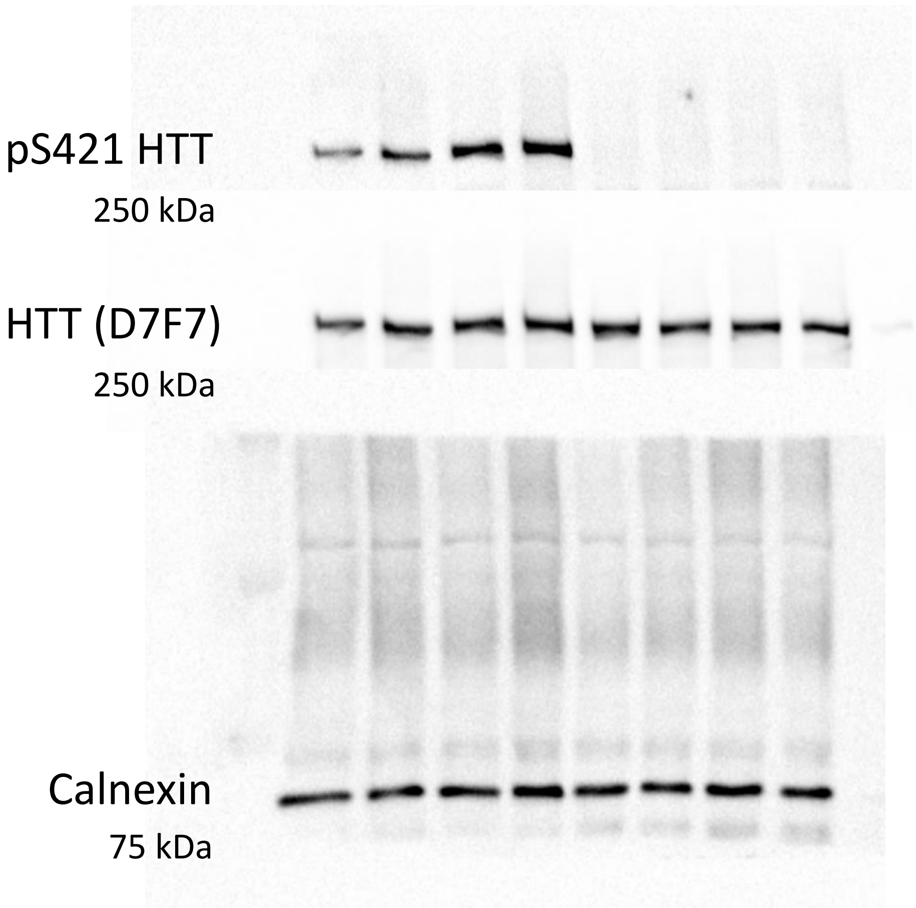

Full unedited gel for Figure EV3B

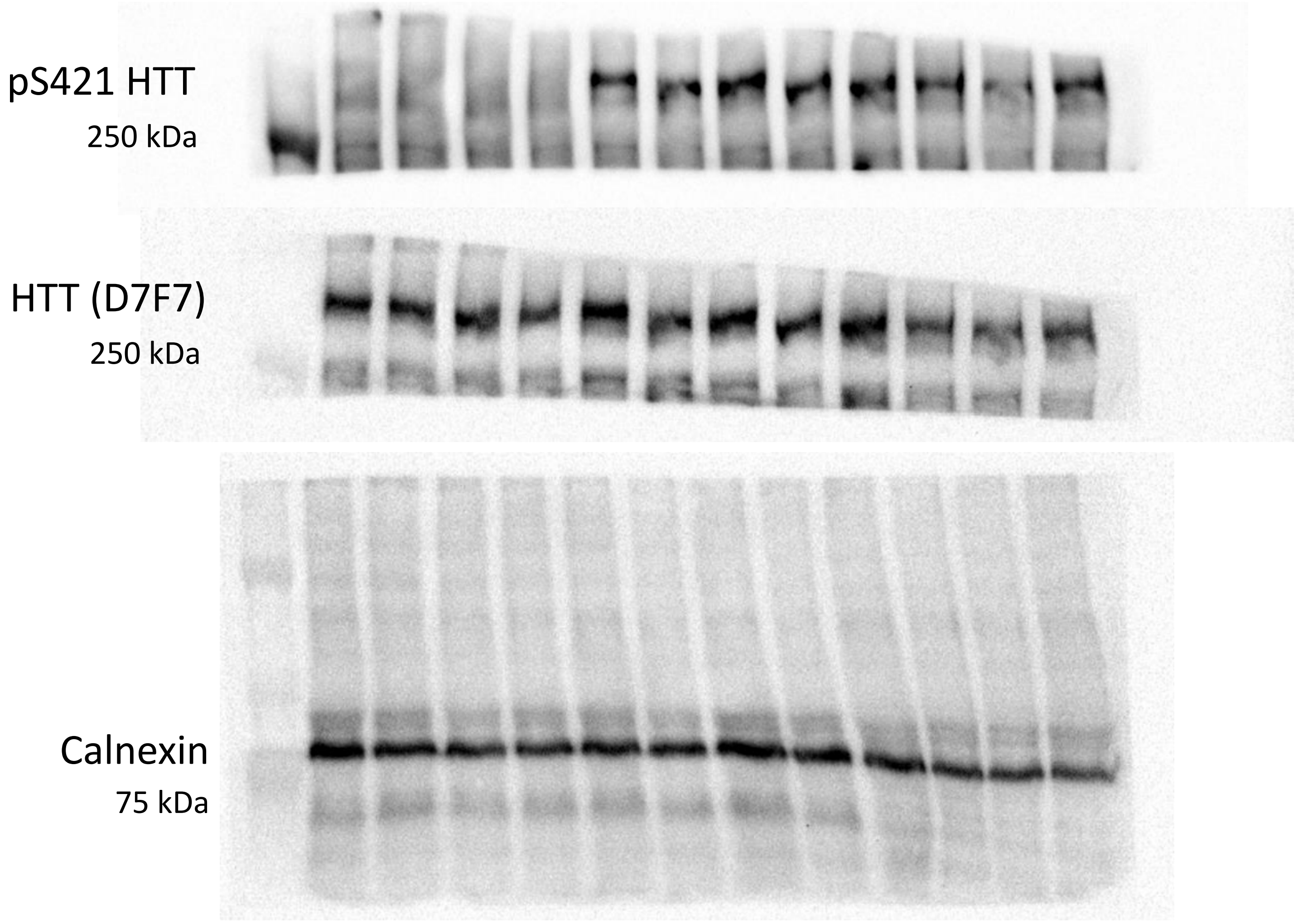

Full unedited gel for Figure EV3C

pS421 HTT

250 kDa

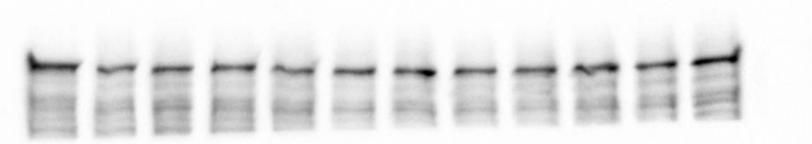

Calnexin

75 kDa kDa

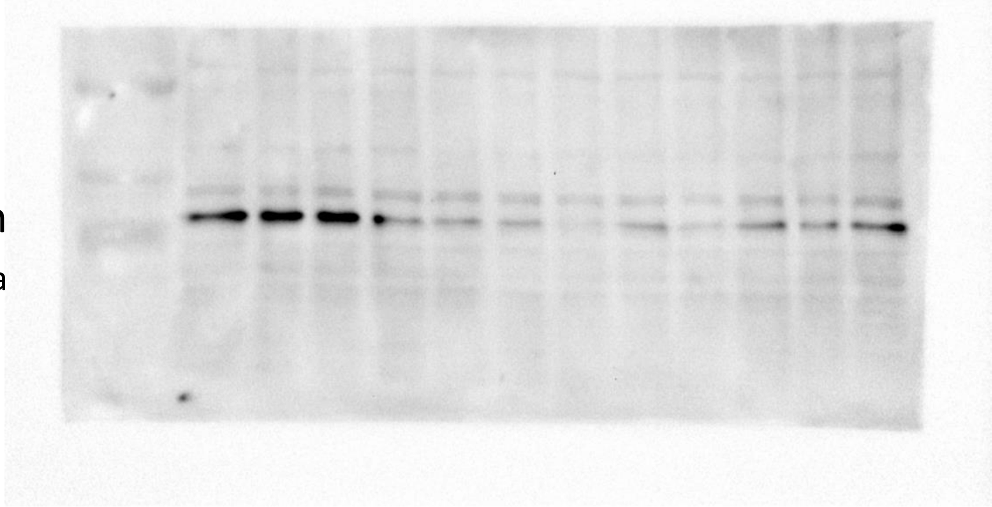

Mecp2

50 kDa

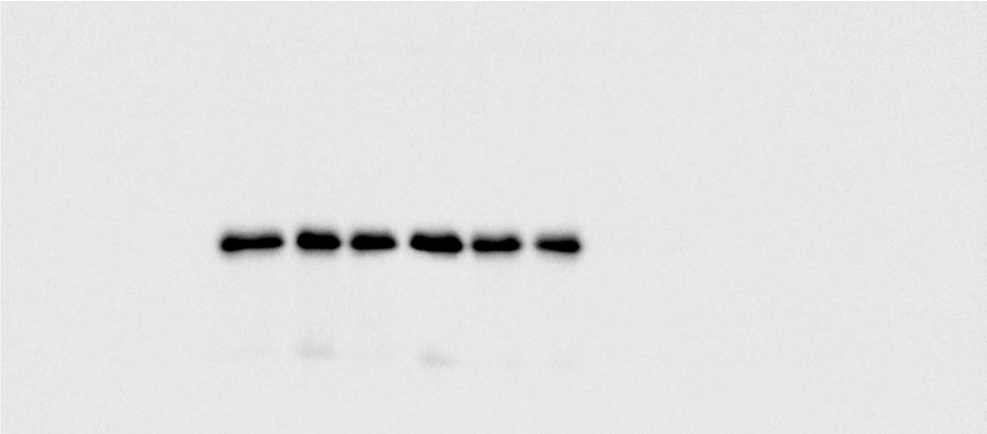

Supplement: Supplementary file 5 — Source Data for Expanded View [file EMMM-12-e10889-s007.zip › EV_source_data/Figure_EV3_source_data.pdf]
