# Supplementary figures and images for: Huntingtin phosphorylation governs BDNF homeostasis and improves the phenotype of Mecp2 knockout mice
Source: EMBO Mol Med. 2020 Jan 8;12(2):e10889. doi: 10.15252/emmm.201910889 (PMC7005633; doi:10.15252/emmm.201910889)

Full unedited gel for Figure 3A

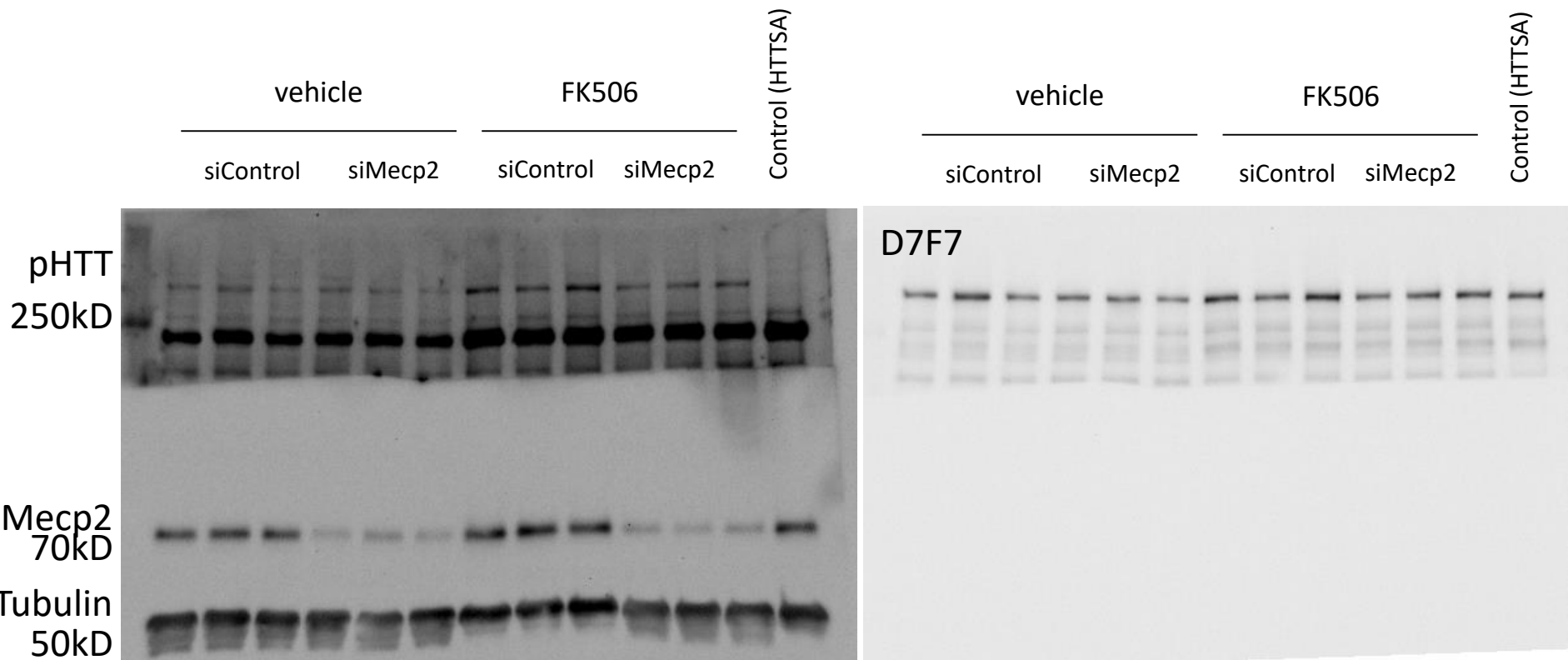

Supplement: Supplementary file 7 — Source Data for Figure 3 [file EMMM-12-e10889-s005.pdf]

Full unedited gel for Figure 4A

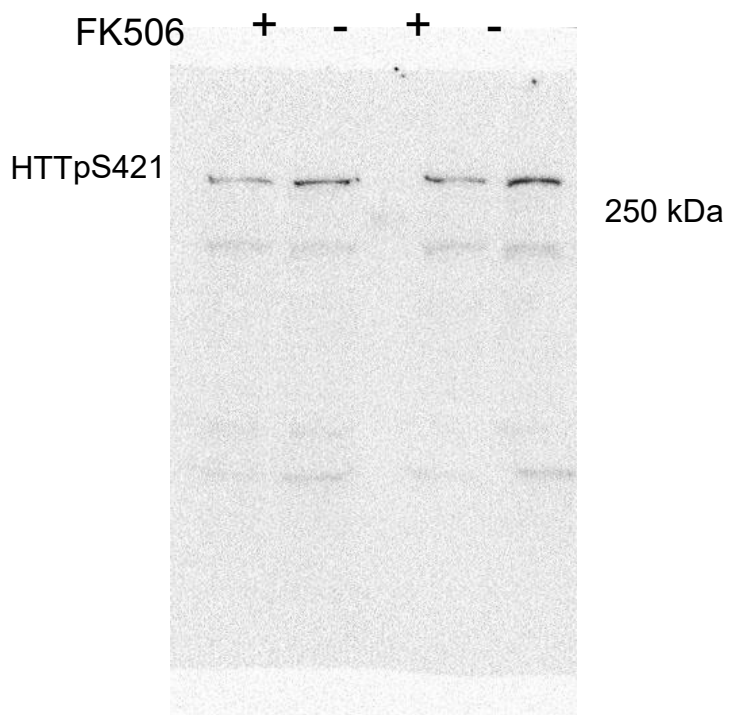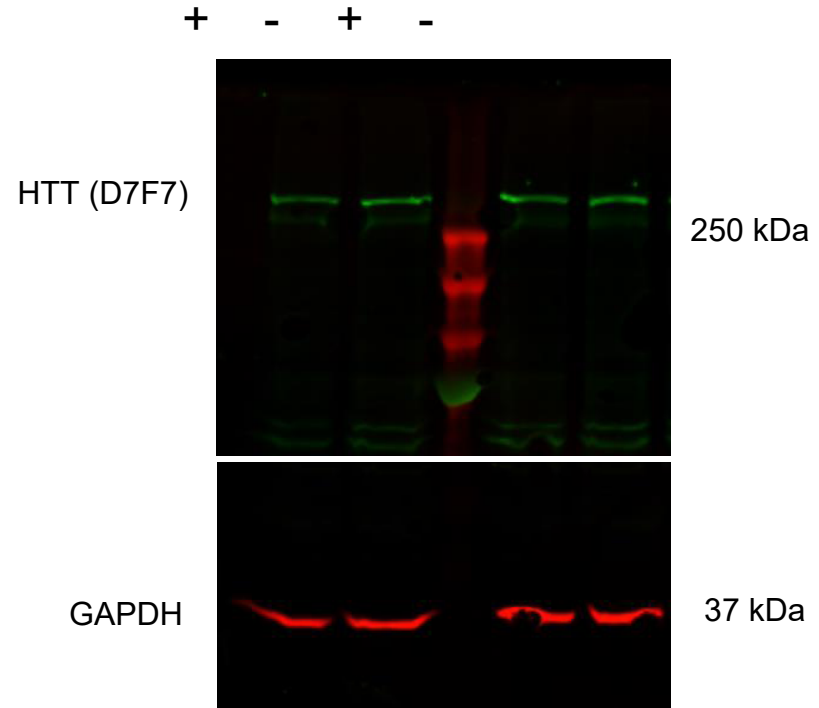

Supplement: Supplementary file 8 — Source Data for Figure 4 [file EMMM-12-e10889-s006.pdf]
